# A scoping analysis of the aspects of primary healthcare physician job satisfaction: facets relevant to the Indonesian system

**DOI:** 10.1186/s12960-019-0375-3

**Published:** 2019-05-30

**Authors:** Chatila Maharani, Hanevi Djasri, Andreasta Meliala, Mohamed Lamine Dramé, Michael Marx, Svetla Loukanova

**Affiliations:** 1 Heidelberg Institute of Global Health, University Hospital Heidelberg, Heidelberg University, Heidelberg, Germany; 2 Department of Public Health, Universitas Negeri Semarang, Semarang, Indonesia; 3 Faculty of Medicine, Public Health and Nursing, Universitas Gadjah Mada, Yogyakarta, Indonesia; 4 Success in Africa, Conakry University Medical Faculty based think tank, Conakry, Guinea; 5 Department of General Practice and Implementation Research, Medical Faculty, University Hospital Heidelberg, Heidelberg University, Heidelberg, Germany

**Keywords:** Health system reform, Job satisfaction, Physician, Primary care

## Abstract

**Background:**

Although there is extensive literature on the different aspects of physician job satisfaction worldwide, existing questionnaires used to measure job satisfaction in developed countries (e.g., the Job Satisfaction Scale) do not capture the aspects specific to Indonesian primary healthcare physicians. This is especially true considering the 2014 healthcare system reform, which led to the implementation of a national social health insurance scheme in Indonesia that has significantly changed the working conditions of physicians. Therefore, the current study aimed to identify aspects of primary care physician job satisfaction featured in published literature and determine those most suitable for measuring physician job satisfaction in light of Indonesia’s recent reforms.

**Methods:**

A scoping literature review of full-text articles published in English between 2006 and 2015 was conducted using the PubMed, Psycinfo, and Web of Science databases. All aspects of primary care physician job satisfaction included in these studies were identified and classified. We then selected aspects mentioned in more than 5% of the reviewed papers and identified those most relevant to the post-reform Indonesian context.

**Results:**

A total of 440 articles were reviewed, from which 23 aspects of physicians’ job satisfaction were extracted. Sixteen aspects were deemed relevant to the current Indonesian system: physical working conditions, overall job satisfaction, patient care/treatment, referral systems, relationships with colleagues, financial aspects, workload, time of work, recognition for good work, autonomy, opportunity to use abilities, relationships with patients, their families, and community, primary healthcare facilities’ organization and management style, medical education, healthcare systems, and communication with health insurers.

**Conclusion:**

Considering the recent reforms of the Indonesian healthcare system, existing tools for measuring job satisfaction among physicians must be revised. Future research should focus on the development and validation of new measures of physician job satisfaction based on the aspects identified in this study.

## Background

Primary healthcare (PHC) physicians may be considered the “gatekeepers” of the healthcare system in many countries, as they are often the first point of contact for patients seeking care or referrals to specialists or hospitals [1–3]. Job satisfaction of PHC physicians was found to profoundly influence the overall quality of the medical care provided [4], their risk of burnout [5, 6], and their willingness to remain in the PHC field [7]. In the present study, job satisfaction is defined as the way PHC physicians feel about their work [8]. This is influenced by many factors, such as individuals’ personality traits, their social environment, including work relationships, and workplace characteristics such as organizational values, working hours, workload, and income [8–11].

Existing literature on physician satisfaction has been showing increased recognition of the numerous difficulties that PHC physicians face in developed countries including workforce shortage, decreasing interest in the profession, and increased desire to retire early [11–14]. Several studies examining job satisfaction among physicians have been conducted following healthcare system reforms in countries such as the United Kingdom, Canada, Norway, China, Taiwan, and Iran [9, 15–20]. For example, Taiwan established a national health insurance program in 1995 and achieved a 99.6% coverage by 2015 [21]. However, Taiwanese PHC physicians were dissatisfied with the program due to the subsequent decline in revenue, increased working hours, unstable regulations, and the complicated claims system [22]. Similarly, in China, reforms applied to implement universal health coverage by 2020 have caused the Chinese healthcare staff to feel overworked and less autonomous, with their income lowered due to the implementation of an essential drug list preventing them from generating additional income from over-prescribing [23]. Iran implemented a family physician program in 2004 [20], which made thousands of family physicians available to the general public resulting in a tenfold increase in the patient-physician ratio [24]. As a result, physician satisfaction scores in one province in the country decreased because of increased job contract insecurity [20].

In Indonesia, PHC is provided by general physicians in government-owned healthcare centers and private healthcare facilities. There are over 9754 district-level health centers [25] nationwide, with various auxiliary health centers located in some sub-districts. Secondary and tertiary healthcare is provided by public and private hospitals in every city [26]. Before the implementation of the national health insurance scheme, *Jaminan Kesehatan Nasional* (JKN), most health service costs were out-of-pocket. Only health centers and a small number of select private PHC physicians had contracts with *PT. Askes*, an organization that provided health insurance for civil servants. Following the reform on January 1, 2014, the government began encouraging Indonesians to register as JKN members. This led to the establishment of far more PHC facilities in contract with *Badan Penyelenggara Jaminan Sosial Kesehatan* (BPJS for Health), a health insurance organization that replaced *PT. Askes* following the implementation of JKN. As a result, the payment system has changed from out-of-pocket to social insurance-based payments.

An important factor influencing physician job satisfaction is the healthcare payment model or financing system [17]. In Indonesia, the healthcare financing system was radically altered by the JKN, changing from a retrospective, fee-for-service (FFS) model to a prospective method based on capitation funding in PHC and diagnosis-related groups (DRGs) in secondary and tertiary care [27]. DRGs are a form of prospective payment based on diagnosis packages; under the capitation model, the income of the PHC facilities is calculated per healthcare scheme participant multiplied by the number of participants registered in those facilities. Thus, PHC facilities must manage their income to fund not only curative and rehabilitative care and essential medical treatment, but also preventive and promotive care [28]. This has led to an increase in cost containment practices affecting the income and the degree of professional autonomy of the physicians [29].

Moreover, with the implementation of clinical pathways and a national formulary, physicians must now comply with clinical practice guidelines more than ever before. PHC service has changed from physician-centered to patient-centered care and from individual-based to team-based services. Healthcare facilities’ eligibility for resources (e.g., practice legalization, human resources, medical facilities) is now assessed by one insurer, BPJS for Health, through a credentialing and evaluation process [30]. Therefore, following the implementation of the JKN, the working culture of PHC has also changed from activity-based to performance-based. The health care reform has also decreased the number of obstetric deliveries attended by traditional birth attendants and increased healthcare utilization, including use by impoverished and near-impoverished people [30–32]. Overall, the system, culture, and patients’ preferences have changed [29], which has significantly impacted physician satisfaction [33].

There are many frameworks for measuring job satisfaction, such as the facets/aspects model, discrepancy model, and steady-state theory [8]. Herzberg’s theory is often mentioned when considering in job satisfaction theories. In the current literature, the theory is included under the umbrella of motivation theory, while a motivation theory, Maslow’s hierarchy of needs, is used as a foundation of job satisfaction theories [34]. The discrepancy model describes how people compare their feelings about their current job to an ideal job, while the steady-state theory posits that people have a baseline level of job satisfaction, which rises or falls relative to that level. However, in this study, we have relied on the facets/aspects model, which involves breaking jobs down into various aspects and evaluating the satisfaction with each aspect [8]. This model has been widely used in studies employing multidimensional instruments [35] such as the Job Satisfaction Scale (JSS) [36]. The latter is one of the most relied-upon tools, designed to measure numerous job aspects relevant to employee satisfaction in the human service sector [37]. The tools measure aspects such as the amount of variety in a job, opportunities to use one’s abilities, freedom of working methods, the extent of responsibilities, physical working conditions, hours spent working, income, recognition for work, relationship with colleagues, and overall job satisfaction. However, it does not capture some of the important job aspects specific to the PHC facilities.

Most research on PHC physician satisfaction in Indonesia has focused on one aspect, the satisfaction with the capitation system, without considering other aspects impacted by the healthcare reforms [38–40]. One study did, however, examine satisfaction with capitation, remuneration, patient numbers, service standards, and working environments following the implementation of JKN [41]. Nonetheless, existing questionnaires do not cover specific aspects for PHC physicians or of the current Indonesian reform. Considering the narrow scope of studies focusing on PHC physician satisfaction following the healthcare reform in Indonesia, it is necessary to determine which aspects of job satisfaction are most relevant post-reform. Therefore, the present study aimed to address the following questions:

1. Which aspects of PHC physician satisfaction were measured in previously published studies?
2. Which aspects are relevant for measuring job satisfaction among PHC physicians in Indonesia under the current health reforms?

## Methods

### Search strategy

A scoping review was conducted using the PubMed, Psycinfo, and Web of Science databases. We systematically searched literature using terms with the same meaning as “physician,” “primary healthcare,” and “satisfaction,” with other words combined using Boolean operators. We did not include “NOT patient/family/parent satisfaction,” because such a search would have overlooked articles examining the relationship between PHC physician satisfaction and patient, family, and parent satisfaction.

**Fig. 1:**
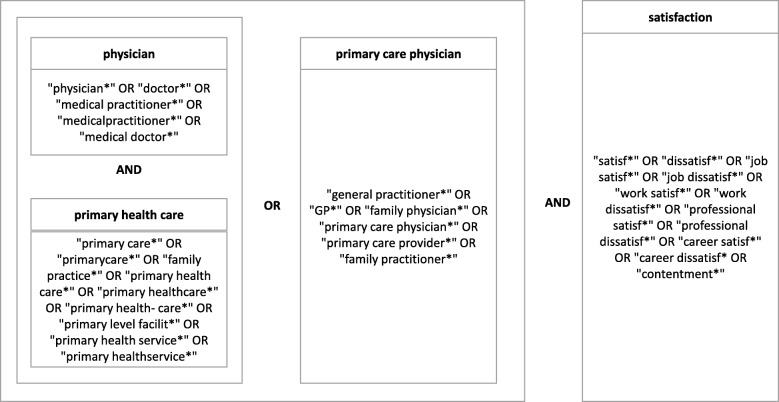
A mind map of the keywords and search strategy

Figure 1 shows a mind map of the keywords and search strategy.

The search was conducted in November 2016 and was limited to English articles published between January 2006 and December 2015.

### Review process

#### Inclusion and exclusion criteria

We included studies on job satisfaction that featured PHC physicians as a sample population and excluded studies that only considered other healthcare professionals such as hospital physicians, nurses, and midwifes. We included articles wherein job satisfaction was a primary or secondary outcome in order to keep the essential aspects of job satisfaction at the primary and secondary level. Both quantitative and qualitative studies were considered in the review.

Studies were excluded if they focused on general practitioners (GPs), medical students, or GPs working in hospitals because such professionals work in different environments than PHC physicians. Study protocols, systematic literature reviews, and questionnaire development articles were also excluded.

The review process had six stages. First, articles were identified through database searches that highlighted date of publication, language, and duplicate articles. Second, we evaluated the relevance of the studies by reviewing their abstracts. Third, we screened the full texts of the remaining articles for eligibility, and fourth, we reviewed the eligibility of these articles.

As the fifth step, we synthesized the various aspects of PHC physician job satisfaction in the selected articles. We identified the aspects of job satisfaction in each article by reading the articles in full, focusing on the methods, results, discussion, and appendices. For articles that did not directly discuss the specific aspects that were measured and reported only the general job satisfaction, we reviewed the questionnaires used in the studies. If the questionnaires were not available, we considered it an overall job satisfaction measure (with overall satisfaction as one of the aspects of physician satisfaction). We then listed all the identified aspects and categorized them based on previously defined aspects in existing questionnaires such as the JSS. When aspects were too specific, we broadened their scope. For example, “future payment prediction” and “balance between income and workload” were grouped into a “financial” category. Additionally, “communication with referral destination” and “variety of local specialists” were merged into a “referral” category. We then counted the number of articles that mentioned each aspect of job satisfaction and calculated the percentage of the total number of articles reviewed that mentioned each aspect.

In the sixth and final step, we identified aspects of job satisfaction relevant to PHC in Indonesia using selection criteria that focused on the aspects of job satisfaction that were mentioned by more than 5% of the articles and were relevant to the current Indonesian healthcare reform. The relevance of these facets to Indonesian PHC is justified in the discussion section of this review. The articles were also classified according to publication year and country in which the research was conducted in order to identify the publication trends in job satisfaction studies.

## Results

**Fig. 2:**
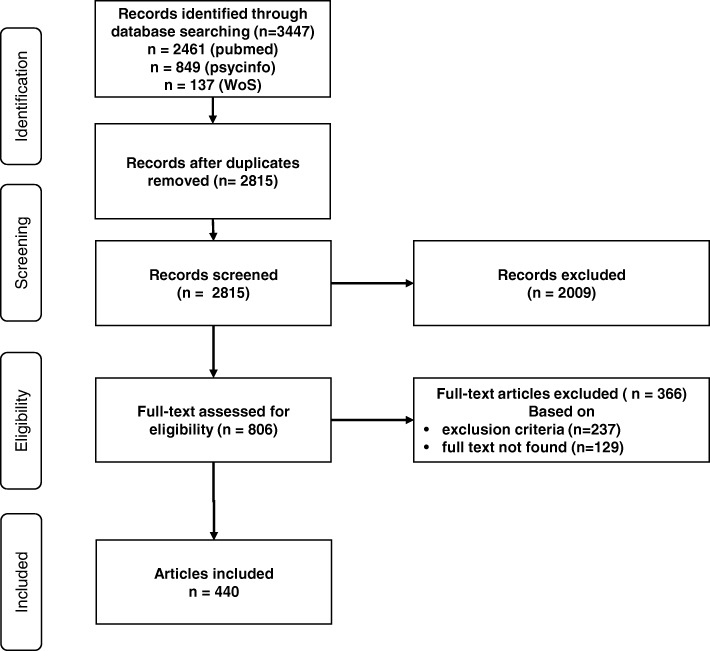
PRISMA 2009 flow diagram of article selection process

We retrieved 3447 articles that met the publication date and language criteria. We checked for duplicate records automatically using Endnote software as well as manually. After duplicate records were removed, 2815 abstracts were reviewed. We excluded 2009 articles via abstract review, 129 via full-text review, and 237 through a second full-text review, leaving 440 articles for the analysis. We extracted the aspects of job satisfaction from those articles. Figure 2 shows the article selection process.

**Fig. 3:**
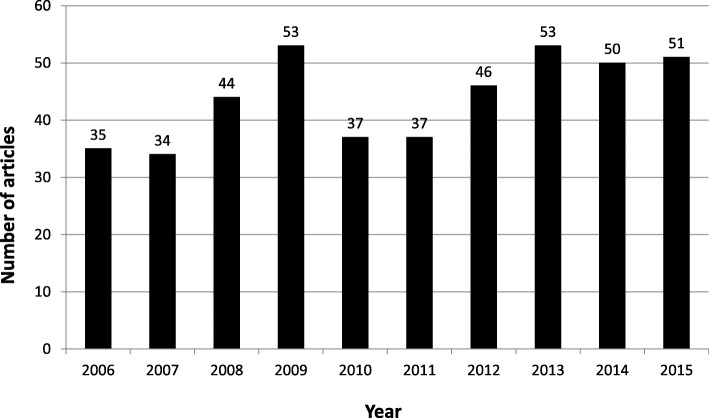
Published articles on job satisfaction in PHC by year

We observed a relatively stagnant trend in PHC physician satisfaction studies over time: the number of published studies increased from 35 in 2006 to 51 in 2015, with the number of published studies peaking at 53 articles in 2009 and 2013. Most studies were conducted on PHC physicians in developed countries, particularly the United States of America (35.45%), Canada (10.91%), the United Kingdom (9.32%), and Australia (7.95%). Multi-country studies were featured only in 2.50% of the reviewed articles and discussed between 2 and 25 countries. Few studies were conducted on countries that underwent recent healthcare system reforms to implement UHC, such as China, Taiwan, and Iran. Figure 3 shows the number of articles on PHC physician satisfaction published annually (2006–2015), and Table 1 displays the distribution of articles by country focus.

**Table 1:** Distribution of studies by country

Country	Number of articles (*n* = 440)
The United States of America	156 (35.45%)
Canada	48 (10.91%)
The United Kingdom	41 (9.32%)
Australia	35 (7.95%)
Netherland	14 (3.18%)
Norway	13 (2.95%)
Multiple countries^a^	11 (2.50%)
Germany	11 (2.50%)
Spain	9 (2.05%)
China	8 (1.82%)
France	7 (1.59%)
Ireland	6 (1.36%)
Denmark	6 (1.36%)
New Zealand	5 (1.14%)
Israel	5 (1.14%)
Iran	5 (1.14%)
Other countries^b^: Finland, Sweden, Switzerland, Belgium, Croatia, Hungary, Italy, Portugal, South Africa, Taiwan, Turkey, Austria, Egypt, Malaysia, Saudi Arabia, Singapore, Afghanistan, Brazil, Colombia, India, Lithuania, Moldova, Mongolia, Nigeria, Pakistan, Rep. Macedonia, Sri Lanka, Tanzania, UAE, Uganda	60 (13.64%)

^a^The studies featured analyses of multiple countries

^b^One to four studies were conducted in each country

We identified 23 aspects of PHC physician job satisfaction. Table 2 shows all 23 aspects, their definitions, reference frequencies across articles, and example articles.

**Table 2:** Aspects of PHC physician job satisfaction, definitions, percentage distributions, and article examples

No.	Aspect	Definition	Percent^a^	Examples^b^
1	Physical working conditions	Satisfaction with facility resources (e.g., medication, technology, laboratory and other diagnostic equipment) and practice location (e.g., practice workspace; geographical location; urban, rural, or remote area; whether physician lives in practice location, weather)	32.50	Satisfaction with laboratory equipment [42], electronic health records (EHR) [43], electronic patient files [44], workplace-practice location [9], building or room for practice [45], community and living near the practice location [46]
2	Overall job satisfaction	Physicians’ level of general satisfaction	30.91	Overall job satisfaction [7]
3	Patient care/treatment	Satisfaction with treatment model/program, therapy/medication, care provision for some diseases, promotive and preventive programs, clinical guidelines, supporting tools for diagnosis, own skill in treating patients’ health problems, presence of clinical problems, availability of clinical information, organization of specific therapeutic procedures related to therapy, and quality of care	21.59	Satisfaction with Geriatric Care Model for managing frail and older people with chronic diseases at home by a team in the Netherlands [47]; satisfaction with a Guided Care model for treating older patients with chronic diseases by a team in Baltimore-Washington, DC [48]; ability to treat mental diseases [49], and promotive and preventive programs for people with Intellectual Disabilities [50].
4	Referral	Satisfaction with referral system, access to referrals, referral destination (other physicians, specialists, hospitals), relationship and communication with referral destination, facilities of referral destination, counter-referral system, waiting time for referral, variety and quality of referral destination in the area, and recommendation or outcome of referral destination	18.64	Satisfaction with the relationship with a hospital [51]
5	Relationship with colleagues	Satisfaction with the relationship with colleagues (other physicians, medical and non-medical staff) or team members, availability, and capability of co-workers	14.55	Satisfaction with the relationship with colleagues, including subordinates and nurses [9], and with the availability of locum tenens [51]
6	Financial	Satisfaction with current income, reward system, reimbursement from insurance, balance between income and workload, prediction of future income, market availability, and other financial benefits (e.g., health insurance, retirement, vacation, education, financing of continuing medical education [CME]).	12.95	Satisfaction with balance between income and workload [52] and with Medicare/Medicaid reimbursement [46]
7	Workload	Satisfaction with number of patients, work pressure, and variety of work (e.g., administration, promotion, management, teaching, and research)	12.27	Satisfaction with promotion tasks, management, teaching [6], and administrative work for documentation [6, 46]
8	Time of work	Satisfaction with work time, schedule, time management, time for taking care of patients, time outside of work (e.g., personal time, vacation, sick leave)	11.36	Satisfaction with time outside of work (sick leave and vacation or leisure time) [9]
9	Autonomy/freedom to choose own method of work	Satisfaction with the extent to which physicians have autonomy to choose their method of work	9.32	Satisfaction with autonomy [11] and self-decisions in the job [52]
10	Recognition for good work	Satisfaction with the extent to which physicians are appreciated by patients, colleagues, senior staff, managers, and the community	9.09	Satisfaction with appreciation by patients, colleagues, managers, and the community [53]
11	Opportunity to use abilities	Satisfaction with the extent to which physicians can use their abilities in their work	7.27	Satisfaction with the opportunity to improve work capacity [52]
12	Relationship with patients, families, and communities	Satisfaction with relationships with patients, patients’ family, and community, communication with the patient and families, patient and families involvement, attention, and satisfaction	6.59	Satisfaction with physicians’ contact with patients [11], consultations with parents, and family interventions [6]
13	PHC facility organization and management style	Satisfaction with the organization of their practice, such as the administration and management, vision, relationship with managers, managers’ competence, and the financial aspects of the organization.	5.91	Satisfaction with the management system in the workplace and physicians’ immediate boss [54]
14	Education	Satisfaction with previous education (e.g., residency training) and learning opportunities (training, congress, library access, professional consultation, CME).	5.00	Satisfaction with training residency [55] and learning or CME opportunities [10, 11]
15	Practice in medicine	Satisfaction with practicing medicine and choosing their specialty	3.18	Satisfaction with practicing medicine [56] and the selection of specialty [57]
16	Promotion	Satisfaction with opportunities for a promotion or career development	2.50	Satisfaction with the transparency of promotion path [58] and opportunity for promotion [54]
17	Personal life	Satisfaction with their own life including balance between job and personal life	2.50	Satisfaction with balance between job and private life [52, 58]
18	Job security	Satisfaction with the stability of their work	1.82	Satisfaction with job security [54] and stability of work [52]
19	Healthcare system/healthcare regulation/law	Satisfaction with healthcare system (in general and for specific diseases), regulations regarding practice of medicine, and malpractice environment.	1.59	Satisfaction with malpractice environment and administrative requirements for the practice of medicine [57], healthcare system for mental diseases [59] and for people with intellectual disabilities [50]
20	Spouse and family satisfaction	Physicians’ satisfaction with spouse and family satisfaction, specifically location, jobs for spouses, children’s education, social relationships, and quality of family life	1.36	Satisfaction with spouses’ satisfaction with practice location, physicians’ work life, spouses’ career, and closeness of extended family [46]
21	Stress-related work	Satisfaction with their level of work stress	0.91	Satisfaction with the level of work stress [58]
22	Health insurer	Satisfaction with the organization of the health insurance (e.g., communication)	0.23	Satisfaction with communication [60]
23	Job performance	Satisfaction with their job achievement	0.23	Satisfaction with their job performance [56]

^a^The number of articles in each aspect divided by the total number of articles (440 in the review

^b^Example of aspects in the reviewed articles

## Discussion

Of the 23 aspects of PHC physician job satisfaction, several were not featured in the JSS questionnaire despite the importance of the aspects such as referral systems, relationships with patients and their families, patient care and treatment, and healthcare and health insurer systems at the systemic level. Our results seem to confirm that while the JSS is suitable for measuring job satisfaction in the human services sector [37], it is not entirely appropriate for measuring satisfaction among PHC physicians.

### Job satisfaction facets relevant to Indonesian PHC

We identified 13 aspects of PHC physician satisfaction that are relevant to the Indonesian context and were mentioned in over 5% of articles: physical working conditions, overall job satisfaction, patient care/treatment, referral systems, relationships with colleagues, financial characteristics, workload, time of work, degree of autonomy, recognition for good work, opportunities to use abilities, relationships patients and their families, and PHC facility organization and management style. Furthermore, because changes in the healthcare system shape changes in physician satisfaction, we identified three aspects of PHC physician satisfaction relevant to developing countries and countries undergoing healthcare reforms: medical education, healthcare system type, and health insurance organizations. For example, health insurance organizations have played a key role in healthcare reforms in South Korea [19], and gaps in the medical education system in developing countries can lead to a lack of competency among physicians [61]. The 16 aspects are discussed in detail in the following paragraphs.

*Physical working conditions* included practice location (i.e., in urban, suburban, and rural areas), practice workspace, and facility resources. While the Indonesian government has established healthcare centers and placed more medical residents in island areas and impoverished regions, these areas remain underdeveloped and have limited access to PHC [62]. The physical conditions of these healthcare centers also vary widely. In 2013, 64% of centers were in good physical condition, while 26% had minor physical flaws, 9% had sustained severe physical damage, and 0.52% were completely damaged [63]. Moreover, the availability of electricity and clean water in healthcare centers also varies: a 2011 study by the Ministry of Health found that over a 24-h period, electricity availability in one healthcare center ranged from 35.6 to 99.8%, while clean water availability ranged from 29.5 to 89% [26]. A study of three cities in the East Java Province, an area with one of the highest levels of development in Indonesia, found that 90.8% of physicians were satisfied with their working conditions [64]. This suggests that lower job satisfaction may be found in areas with poor physical working conditions.

Indonesian healthcare system currently operates a tiered referral system that was not optimally implemented before the JKN reforms [26, 27]. Under the FFS system that existed prior to the reforms, many patients visited specialists directly and either skipped visits with PHC physicians entirely or only asked for referral letters from PHC physicians without obtaining treatment. As a result, PHC physicians felt that patients did not appreciate them. Moreover, the lack of facilities forced PHC physicians to refer patients elsewhere [39, 40], which prevented the physicians from fully exercising their abilities. Thus, *recognition of good work* and *opportunities to use abilities* are important aspects of PHC physician job satisfaction that should be considered under the current healthcare system.

Under JKN, patients are encouraged to follow the strictly tiered *referral system* made possible by new regulations for payment eligibility by the BPJS for Health, except in the case of emergencies [65]. Physicians must treat patients in accordance with Indonesian competency standards and clinical practice guidelines for physicians in PHC facilities [66–68]. When necessary, PHC physicians can refer patients to C or D hospitals (i.e., secondary care providers); when higher specialization is needed, secondary care providers can refer patients to A or B hospitals (tertiary care providers). Despite these new regulations, there are many direct referrals from PHC physicians to A hospitals and many cases are treated in hospitals despite falling under the scope of PHC physician care [69]. The referral rates during the *PT. Askes* and JKN eras were 16% and 12.5%, respectively. Both referral rates are higher than the national standard of 10% [40, 70].

JKN introduced several new programs providing guidelines for PHC physicians in *patient care and treatment*, such as the Management of Chronic Diseases Program for type 2 diabetes and hypertension, as well as the counter-referral, home visits, and health screening programs. Additionally, Indonesian PHC physicians typically work with *colleagues and healthcare professional networks* (e.g., networks of other physicians, dentists, nurses, midwives, pharmacists, and administrative staff). When PHC facilities cannot provide basic services such as immunization and family planning, PHC physicians can refer patients to other PHC facilities using these networks.

*Financial issues* are increasingly important to PHC physician satisfaction in Indonesia [38–41]. Before 2014, PHC clinics used the FFS system, where employed PHC physicians were paid by the PHC facility owners at a fixed salary and/or based on the number of patients examined. During this time, only a small number of PHC facilities had contracts with *PT. Askes* and used the capitation system. Physicians were somewhat dissatisfied with the capitation system used by *PT. Askes* [38–40]. Under JKN, PHC facilities are paid mainly based on capitations [27]. There is also additional income from capitation for healthcare center physicians [71]. For PHC physicians who work as employees in PHC clinics, income payments are dependent on the manager or owner of their respective PHC facilities. The management style of PHC facility leadership is an important aspect of the job satisfaction for the employed PHC physicians. Research conducted in a district in the East Java province showed that the leadership styles of healthcare center managers are associated with employee performance [72]. Because of this relationship, there is a need to measure the physician satisfaction with *PHC facility organization and management style*.

Due to the relatively low amount of nominal capitation, the capitation system may lead physicians to perceive that they have less *autonomy* by limiting their ability to treat patients [40].

Since the number of PHC facilities is constantly rising—from 13 209 facilities in 2012 to nearly 19 969 facilities in 2015—the working conditions for many PHC physicians are changing [73, 74]. The ratio of physicians per 100 000 inhabitants remains low in Indonesia, at about 16.04 (10.95–39.18 by province) [25]. There has also been an increase in the number of patients visiting PHC centers, from 61.7 million in 2014 to 100.6 million in 2015 [74, 75]. Thus, PHC physicians face an increasingly high *workload*. Apart from patient care duties, PHC physicians working in healthcare centers often perform administrative and bureaucratic tasks such as holding meetings regarding their areas of responsibility in a given district. Various new tasks have also been introduced, such as promotion and preventive services, basic immunization, and family planning programs. A study conducted on the island of Sulawesi revealed that some healthcare workers were less satisfied because of a lack of a sense of reward and high workload [76].

*Time of work* is another aspect of job satisfaction that was revealed in the current results. Indonesian physicians are allowed to practice at a maximum of three facilities [77]. Increasingly more physicians work as civil servants in public hospitals or healthcare centers in the morning and private clinics or solo practices in the afternoon [78]. Additionally, physicians providing inpatient care in healthcare centers or PHC clinics have on-call duty (i.e., work that takes place outside of their normal hours). Many physicians also provide health services outside of their practice schedules, typically to neighbors and relatives, due to the Indonesian cultural practices.

While *relationships with patients, their families, and community* members were only mentioned in 6.59% of the articles reviewed, after the introduction of the JKN, PHC services have become more patient-oriented through the gatekeeper concept [79]. PHC physicians are the first point of contact for patients and their families seeking healthcare. Therefore, physicians’ satisfaction with their relationship with patients should be measured. There have been reports of violence against PHC physicians by patients’ family members [80], suggesting that family-physician relationships are important facets to consider. In addition, one study found that 81% of the community of JKN participants were satisfied with PHC, hospital, and BPJS for Health services [81].

The Indonesian healthcare system still struggles with specifying the competencies of healthcare workers [82]. Indonesian PHC physicians were found to independently manage only one third of the cases that fall into the range of ailments that must be treated by PHC physicians, [83]. This may be because only about 22% of medical faculty have an A status—the highest accreditation status [28]. Accordingly, even though *medical education* was mentioned in only 5.00% of articles, physicians’ satisfaction with it should be considered when measuring job satisfaction.

The *healthcare system* and *health insurer* aspects of PHC physician job satisfaction were mentioned in only 1.59% and 0.23% of the articles, respectively. Similarly to Indonesia, South Korea and Taiwan both have implemented single-payer insurance systems and examined physicians’ satisfaction with the healthcare system and health insurer—the BPJS for Health [19, 22, 84, 85]. Single-player systems can lead to greater monopsony power and, by extension, greater purchasing power of the insurers [86]. Thus, measurement of PHC physician satisfaction with the BPJS for Health, the single-payer insurer under JKN, is needed. In 2015, about 74% of PHC facilities’ were satisfied with BPJS for Health [81].

Finally, *overall job satisfaction* was mentioned in 30.91% of articles. While overall job satisfaction is not a specific aspect of the PHC physician job satisfaction, the concept cannot be ignored because it appears to be the second most frequently measured construct. Moreover, by measuring overall job satisfaction, we may be able to develop a general understanding of job satisfaction following the healthcare reform in Indonesia.

## Strengths and limitations

This review extracted aspects of PHC physician satisfaction in studies published worldwide, including qualitative studies. Thus, the results of the present study revealed a number of well-documented aspects of PHC physician job satisfaction. Nevertheless, our reliance on previous questionnaires means that some relevant aspects were not considered. Furthermore, some articles did not report all the items/aspects of these questionnaires in their publications. Additionally, the present review relied only on free access full-text articles or those accessible through the electronic library of Heidelberg University, thereby potentially omitting a large portion of research. Moreover, a potential bias might have resulted from the fact that only one reviewer selected and synthesized the present aspects.

## Conclusion

The job satisfaction measures that comprise currently validated questionnaires such as the JSS are not always appropriate for measuring the job satisfaction of Indonesian PHC physicians. In this study, we identified 23 aspects of PHC physician job satisfaction from published articles and selected 16 facets deemed most relevant to the Indonesian context, enabling the development of accurate and context-specific measures of Indonesian PHC physician job satisfaction. Future research in this field should disaggregate the existing questionnaires by directly contacting the article authors or through other means in order to update the existing measures. Moreover, further research to confirm these aspects should include focus group discussions with Indonesian healthcare experts as well as engage in further quantitative analysis.

## Abbreviations

BPJS: Badan Penyelenggara Jaminan Sosial
CME: Continuing Medical Education
EHR: Electronic Health Record
FFS: Fee-for-Service
GP: General Practitioner
JKN: Jaminan Kesehatan Nasional
JSS: Job Satisfaction Scale
PHC: Primary Healthcare
UHC: Universal Health Coverage

## References

[1] Healy J, Sharman E, Lokuge B (2006). Australia: health system review. Health Syst Transit.

[2] Schäfer W, Kroneman M, Boerma W, van den Berg M, Westert G, Devillé W (2010). The Netherlands: health system review. Health Syst Transit.

[3] Tjerbo T, Kjekshus LE (2005). Coordinating health care: lessons from Norway. Int J Integr Care.

[4] Edward A, Kumar B, Niayesh H, Naeem AJ, Burnham G, Peters DH (2012). The association of health workforce capacity and quality of pediatric care in Afghanistan. Int J Qual Health Care.

[5] Ozvacic Adzic Z, Katic M, Kern J, Soler JK, Cerovecki V, Polasek O (2013). Is burnout in family physicians in Croatia related to interpersonal quality of care?. Arh Hig Rada Toksikol.

[6] Kushnir T, Cohen AH (2006). Job structure and burnout among primary care pediatricians. Work.

[7] Hann M, Reeves D, Sibbald B (2011). Relationships between job satisfaction, intentions to leave family practice and actually leaving among family physicians in England. Eur J Pub Health.

[8] George JM, Jones GR (2012). Understanding and managing organizational behavior.

[9] Wu D, Wang Y, Lam KF, Hesketh T (2014). Health system reforms, violence against doctors and job satisfaction in the medical profession: a cross-sectional survey in Zhejiang Province, Eastern China. BMJ Open.

[10] Shi L, Song K, Rane S, Sun X, Li H, Meng Q (2014). Factors associated with job satisfaction by Chinese primary care providers. Prim Health Care Res Dev.

[11] Behmann M, Schmiemann G, Lingner H, Kuhne F, Hummers-Pradier E, Schneider N (2012). Job satisfaction among primary care physicians: results of a survey. Dtsch Arztebl Int.

[12] Dale J, Potter R, Owen K, Parsons N, Realpe A, Leach J (2015). Retaining the general practitioner workforce in England: what matters to GPs? A cross-sectional study. BMC Fam Pract.

[13] DesRoches CM, Buerhaus P, Dittus RS, Donelan K (2015). Primary care workforce shortages and career recommendations from practicing clinicians. Acad Med.

[14] Pit SW, Hansen V (2014). Factors influencing early retirement intentions in Australian rural general practitioners. Occup Med (Lond).

[15] Fairhurst K, May C (2006). What general practitioners find satisfying in their work: implications for health care system reform. Ann Fam Med.

[16] Kaiser Family Foundation, Commonwealth Fund (2015). Experiences and attitudes of primary care providers under the first year of ACA coverage expansion: findings from the Kaiser Family Foundation/Commonwealth Fund 2015 national survey of primary care providers. Issue Brief (Commonws Fund).

[17] Green ME, Hogg W, Gray D, Manuel D, Koller M, Maaten S (2009). Financial and work satisfaction: impacts of participation in primary care reform on physicians in Ontario. Healthc Policy.

[18] Aasland OG, Rosta J, Nylenna M (2010). Healthcare reforms and job satisfaction among doctors in Norway. Scand J Public Health.

[19] Chen MS, Lee CB (2013). Between professional dignity and economic interests–evidence based on a survey of Taiwan’s primary care physicians. Int J Health Plann Manag.

[20] Jabbari H, Pezeshki MZ, Naghavi-Behzad M, Asghari M, Bakhshian F (2014). Relationship between job satisfaction and performance of primary care physicians after the family physician reform of East Azerbaijan province in Northwest Iran. Indian J Public Health.

[21] National Health Insurance Administration MoHaW, Taiwan. 2015-2016 National health insurance annual report. Taipei (TW). 2015. https://www.nhi.gov.tw/Resource/webdata/30285_1_National%20Health%20Insurance%20in%20Taiwan%202015-2016%20(bilingual).pdf. Accessed 17 May 2019

[22] Lin HC, Chang WY, Tung YC (2003). Factors related to dissatisfaction with the National Health Insurance among primary care physicians in Taiwan.

[23] Zhou XD, Li L, Hesketh T (2014). Health system reform in rural China: voices of healthworkers and service-users. Soc Sci Med.

[24] Takian A, Rashidian A, Kabir MJ (2011). Expediency and coincidence in re-engineering a health system: an interpretive approach to formation of family medicine in Iran. Health Policy Plan.

[25] Ministry of Health (2016). Indonesia health profile 2015.

[26] President of Republic of Indonesia. Presidential regulation on national health system number 72. Jakarta: Republic of Indonesia; 2012. Indonesian.

[27] Coordinating Ministry for Human Development, National Social Security Council, Ministry of Health, Ministry of National Development Planning/National Development Planning Agency, Ministry of Finance, Ministry of State Owned Enterprises, et al. Road map towards National Health Insurance 2012-2019. Jakarta: National Social Security Council; 2012. Indonesian.

[28] World Health Organization Regional Office for South-East Asia (2017). The Republic of Indonesia health system review.

[29] Song Z (2014). Becoming a physician in the age of payment reform. Healthc (Amst).

[30] World Bank (2018). Functional and regulatory review of strategic health purchasing under JKN : purchasing of primary health care under JKN (English).

[31] Teplitskaya L, Dutta A (2018). Has Indonesia’s national health insurance scheme improved access to maternal and newborn health services?.

[32] Rolindrawan D (2015). The impact of BPJS health implementation for the poor and near poor on the use of health facility. Procedia Soc Behav Sci.

[33] Zhang M, Wang W, Millar R, Li G, Yan F (2017). Coping and compromise: a qualitative study of how primary health care providers respond to health reform in China. Hum Resour Health.

[34] Kian TS, Rajah S, Yusoff WFW (2014). Job satisfaction and motivation: what are the difference among these two?. EJBSS.

[35] van Saane N, Sluiter JK, Verbeek JH, Frings-Dresen MH (2003). Reliability and validity of instruments measuring job satisfaction—a systematic review. Occup Med (Lond).

[36] Warr P, Cook J, Wall T (1979). Scales for the measurement of some work attitudes and aspects of psychological well-being. J Occup Organ Psychol.

[37] Spector P (1985). Measurement of human service staff satisfaction: development of the job satisfaction survey. Am J Community Psychol.

[38] Wintera IG (2005). The determinants of community health center doctors satisfaction with capitation payment system of PT. Askes participants at Donggala district, Centre Sulawesi. J Manajemen Pelayanan Kesehatan.

[39] Karyati M. Satisfaction level of family physicians to the capitation payment system of PT. Askes in Medan City. Jurnal Manajemen Pelayanan Kesehatan. 2004;7(2):81–7. Indonesian.

[40] Hendrartini Y (2008). The determinants of GP’s performance on capitation payment. J Manajemen Pelayanan Kesehatan.

[41] Mumtazah R. Analysis of physician satisfaction on capitation system in national health insurance program at primary health service in Banda Aceh City. Banda Aceh: Universitas Syiah Kuala; 2015. Indonesian.

[42] Kairys J, Zebiene E, Sapoka V, Zokas I (2008). Satisfaction with organizational aspects of health care provision among Lithuanian physicians. Cent Eur J Public Health.

[43] Jones CD, Holmes GM, Lewis SE, Thompson KW, Cykert S, DeWalt DA (2013). Satisfaction with electronic health records is associated with job satisfaction among primary care physicians. Inform Prim Care.

[44] Giveon S, Yaphe J, Hekselman I, Mahamid S, Hermoni D (2009). The e-patient: a survey of israeli primary care physicians! Responses to patients’ use of online information during the consultation. Isr Med Assoc J.

[45] Rapport F, Doel MA, Greaves D, Elwyn G (2006). From Manila to monitor: biographies of general practitioner workspaces. Health (London).

[46] Giriyappa P, Sullivan KK (2009). Career satisfaction and retention risk among Wisconsin internists. WMJ.

[47] Muntinga ME, Van Leeuwen KM, Schellevis FG, Nijpels G, Jansen A (2015). From concept to content: assessing the implementation fidelity of a chronic care model for frail, older people who live at home. BMC Health Serv Res.

[48] Marsteller JA, Hsu YJ, Reider L, Frey K, Wolff J, Boyd C (2010). Physician satisfaction with chronic care processes: a cluster-randomized trial of guided care. Ann Fam Med.

[49] Clatney L, Macdonald H, Shah SM (2008). Mental health care in the primary care setting: family physicians’ perspectives. Can Fam Physician.

[50] Lin J-D, Hsu S-W, Yen C-F, Chou Y-T, Wu C-L, Chu CM (2009). Roles of general practitioners in the provision of health care services for people with intellectual disabilities: a national census in Taiwan. J Appl Res Intellect Disabil.

[51] Vanasse A, Scott S, Courteau J, Orzanco MG (2009). Canadian family physicians’ intentions to migrate: associated factors. Can Fam Physician.

[52] Luo Z, Bai X, Min R, Tang C, Fang P (2014). Factors influencing the work passion of Chinese community health service workers: an investigation in five provinces. BMC Fam Pract.

[53] Al Juhani AM, Kishk NA (2006). Job satisfaction among primary health care physicians and nurses in Al-Madinah Al-Munawwara. J Egypt Public Health Assoc.

[54] Chew B, Ramli A, Omar M, Ismail I (2013). A preliminary study of job satisfaction and motivation among the Malaysian primary healthcare professionals. Malays Fam Physician.

[55] Donnelly MJ, Lubrano L, Radabaugh CL, Lukela MP, Friedland AR, Ruch-Ross HS (2015). The Med-Peds hospitalist workforce: results from the American Academy of Pediatrics workforce survey. Hosp Pediatr.

[56] Lepnurm R, Dobson R, Backman A, Keegan D (2007). Factors associated with career satisfaction among general practitioners in Canada. Can J Rural Med.

[57] Aseltine RH, Katz MC, Geragosian AH (2010). Connecticut 2009 Primary Care Survey: physician satisfaction, physician supply and patient access to medical care. Conn Med.

[58] Bagheri S, Janati A, Kousha A, Sadeghi-Bazargani H, Asghari-Jafarabadi M, Farahbakhsh M (2013). Job satisfaction differences between primary health care and treatment sectors: an experience from Iran. Health Promot Perspect.

[59] Beel JV, Gringart E, Edwards ME (2008). Western Australian general practitioners’ views on psychologists and the determinants of patient referral: an exploratory study. Fam Syst Health.

[60] Mrduljas-Dujic N, Kuzmanic M, Kardum G, Rumboldt M (2010). Job satisfaction among medical doctors in one of the countries in transition: experience from Croatia. Coll Antropol.

[61] Al-Shamsi M (2017). Addressing the physicians’ shortage in developing countries by accelerating and reforming the medical education: is it possible?. J Adv Med Educ Prof.

[62] Ministry of Health (2015). Indonesia health profile 2014.

[63] Ministry of Health (2013). Basic data of health centre.

[64] Chotimah N, Kusnanto H. Factors influencing work satisfaction and motivation of family doctor of PT. Askes in providing health services for PT. Askes member in Malang, Madiun, and Kediri, of East Java Province. Jurnal Manajemen Pelayanan Kesehatan. 2000;3(4):171–85. Indonesian.

[65] BPJS for Health (2014). Practical guide of referral system.

[66] BPJS for Health (2014). Primary health facilities handle 144 illnesses deleting “giant heath centre”.

[67] Indonesian Medical Council (2012). Competence standard of Indonesia doctor.

[68] Ministry of Health. Regulation of the Ministry of Health of the Republic of Indonesia number 5 about clinical practice guidelines for physicians in primary health care facilities. Jakarta: Ministry of Health; 2014. Indonesian.

[69] Britton K, Koseki S, Dutta A (2018). Expanding markets while improving health in Indonesia: private health sector market in the JKN era.

[70] BPJS for Health (2018). Health management program reports and financial reports of social health insurance 2017.

[71] Ministry of Health. Regulation of the Ministry of Health of the Republic of Indonesia number 21 about use of capitation fund of National Health Insurance for health services and operational cost support in primary health care facilities owned by regional government. Jakarta: Ministry of Health; 2016. Indonesian.

[72] Chairunnisah R, Nuryadi, Witcahyo E (2014). The correlation between leadership and employee’s work motivation with working performance of public health center in Jember. e-J Pustaka Kesehatan.

[73] PT ASKES (Persero). Annual report 2012: towards transformation era. Jakarta: PT ASKES (Persero); 2013. Indonesian.

[74] BPJS for Health. Financial management report 2015 and financial report 2015 (Audit). Jakarta: BPJS for Health; 2015. Indonesian.

[75] BPJS for Health. Financial management report 2014 and financial report 2014 (Audit). Jakarta: BPJS for Health; 2014. Indonesian.

[76] Anggraeni R, Maidin A, Arifah N (2016). Description of health officer’s satisfaction and the insured of Indonesia national health insurance in South Sulawesi, Southeast Sulawesi and West Sulawesi 2014. J Kebijakan Kesehatan Indonesia.

[77] President of Republic of Indonesia. Law of Republic of Indonesia number 29 about medical practice. Jakarta: Republic of Indonesia; 2004. Indonesian.

[78] Heywood P, Harahap NP, Aryani S (2011). Recent changes in human resources for health and health facilities at the district level in Indonesia: evidence from 3 districts in Java. Hum Resour Health.

[79] BPJS for Health (2014). Practical guide gate keeper concept: health facilities of BPJS for Health.

[80] Saifan DS. Not giving a referral letter, health centre physician was beaten by family patients. Bireuen: Kompas; 2015. Indonesian.

[81] BPJS for Health. Participant and health facilities satisfaction index to BPJS for Health has exceeded target successfully. Jakarta; 2015. https://www.bpjs-kesehatan.go.id/bpjs/index.php/arsip/view/217. Accessed 2 Jan 2015. Indonesian

[82] Luti I, Hasanbasri M, Lazuardi L. Government policy in improving health referral system islands region district in Lingga District Province of Riau Archipelago. Jurnal Kebijakan Kesehatan Indonesia. 2012;1(1):24–35. Indonesian.

[83] Istiono W, Claramita M, Ekawati FM, Gayatri A, Sutomo AH, Kusnanto H (2015). Physician’s self-perceived abilities at primary care settings in Indonesia. J Fam Med Prim Care.

[84] Kim Kye-Hyun, Park Eun-Cheol, Hahm Myung-Il (2012). The Gap Between Physicians and the Public in Satisfaction with the National Health Insurance System in Korea. Journal of Korean Medical Science.

[85] Lee HY, Park SE, Park EC, Hahm MI, Cho WH (2008). Job satisfaction and trust in Health Insurance Review Agency among Korean physicians. Health Policy.

[86] Hussey P, Anderson GF (2003). A comparison of single- and multi-payer health insurance systems and options for reform. Health Policy.

